# The emerging nanomedicine-based technology for non-small cell lung cancer immunotherapy: how far are we from an effective treatment

**DOI:** 10.3389/fonc.2023.1153319

**Published:** 2023-04-27

**Authors:** Lei Peng, Quan Xu, Sui Yin, Ye Zhang, Hao Wu, Yangchun Liu, Liru Chen, Yeji Hu, Jun Yuan, Kai Peng, Qin Lin

**Affiliations:** Department of Thoracic Surgery, Jiangxi Provincial People’s Hospital, The First Affiliated Hospital of Nanchang Medical College, Nanchang, China

**Keywords:** non-small cell lung cancer, immunotherapy, nanomedicine, nanotechnology, combined therapy

## Abstract

Non-small cell lung cancer (NSCLC) is a prominent etiology of cancer-related mortality. The heterogeneous nature of this disease impedes its accurate diagnosis and efficacious treatment. Consequently, constant advancements in research are imperative in order to comprehend its intricate nature. In addition to currently available therapies, the utilization of nanotechnology presents an opportunity to enhance the clinical outcomes of NSCLC patients. Notably, the burgeoning knowledge of the interaction between the immune system and cancer itself paves the way for developing novel, emerging immunotherapies for treating NSCLC in the early stages of the disease. It is believed that with the novel engineering avenues of nanomedicine, there is a possibility to overcome the inherent limitations derived from conventional and emerging treatments, such as off-site drug cytotoxicity, drug resistance, and administration methods. Combining nanotechnology with the convergence points of current therapies could open up new avenues for meeting the unmet needs of NSCLC treatment.

## Introduction

Globally, the prevalence and mortality rates of cancer are increasing, with lung cancer constituting the most frequently diagnosed form of the disease, accounting for 11.6% of all reported cases. In the United States, it is anticipated that approximately 236,740 fresh cases of lung cancer will emerge in 2022, culminating in approximately 130,180 fatalities (1, 2). Lung cancer continues to be the primary cause of cancer-related deaths worldwide, responsible for 18.4% of all cancer mortalities. This results in a substantial societal burden and economic loss (2, 3). Approximately 80% of lung cancer fatalities are attributed to smoking. Other significant risk factors for the development of lung cancer include exposure to radon and asbestos, prolonged and cumulative inhalation of air pollution, particularly emissions of polycyclic aromatic hydrocarbons (PAHs), as well as a personal or familial history of lung cancer (4, 5). Lung cancer can be broadly classified into two main types: non-small cell lung cancer (NSCLC) and small cell lung cancer (SCLC), with NSCLC being the more prevalent type (6). The survival rates for both non-small cell lung cancer (NSCLC) and small cell lung cancer (SCLC) in the metastatic stage are notably low, with a mere 4% 5-year survival rate (7, 8). There are three sub-types of NSCLC, which are classified according to the type of lung cells. These are adenocarcinomas, which arise from abnormal lung cells; squamous cell carcinomas, which arise in the bronchi; and large cell carcinomas, which arise peripherally. They all behave in the same way and respond to treatment in the same way (6). Comprehending the prognosis and attributes of lung cancer enables clinicians to provide the most efficacious therapeutic intervention. An array of therapeutic modalities are available, such as surgical intervention, radiotherapy, chemotherapy, targeted therapy, and immunotherapy, which are prescribed based on the stage of the disease, histological subtype, cellular morphology, and clinical status. However, researchers in clinical practice and clinical trials persistently emphasize the crucial aspects of novel therapies, particularly the profound effectiveness and minimal incidence of adverse events (9–11). A myriad of immunotherapeutics has obtained commendable results in the treatment of various cancers, they also meet some daunting challenges such as low water solubility, poor pharmacokinetic profiles, less absorption, less accumulation in the tumor site, less bioactivity after prolonged circulation, and enhanced immune-mediated off-target toxicity (12, 13). As far as we are aware, nanotechnology has the potential to address these issues by utilizing its advantageous characteristics, thus achieving the expected level of clinical benefits (14–17). Based on a comprehensive understanding of the tumor microenvironment, intelligent and stimuli-responsive nanocarriers are being developed to leverage the advantages of acidic pH, hypoxia, enhanced adenosine-triphosphate (ATP) synthesis, altered redox state of cancer cells, and other relevant factors (18). Nanoparticles have been shown to amplify the advantages of cancer immunotherapy by (1) affording protection to antigens and adjuvants; (2) delivering them simultaneously to the antigen-presenting cells (APCs); (3) reprogramming the microenvironment to resume immune surveillance.

A diverse array of nanoscale drug delivery systems (DDS) are presently being investigated, encompassing liposomes, protein-based nanocarriers, inorganic carriers, and polymeric nanoparticles. The utilization of tailored nanoparticles to manage cancer could represent the next stage in enhancing the effectiveness of conventional therapies, which tend to lack specificity (19–21). With the advent of targeted nanosystems (e.g., functionalized magnetic nanoparticles), targeted DDS platform interventions for NSCLC have shown great promise (22). Targeted functionalized magnetic nanoparticles (MNPs) not only improve the precision of chemotherapy but also result in enhanced pharmacological activity and reduced adverse events. Furthermore, they offer desirable advantages as drug delivery vectors (22, 23), including improved drug stability, drug solubility, and high drug loading capacity (24). A number of peptides and polymers have been added to MNPs to facilitate drug delivery or improve their bio-compatibility (25, 26). Researchers have developed a potential cancer treatment method that targets tumor-related blood vessels by targeting nanoparticle-based avenues to surrogates of angiogenesis, such as receptors for vascular endothelial growth factor (VEGF) or integrins (27, 28). Various nanoparticle-based therapies have been explored as potential treatments for metastatic NSCLC, including albumin-bound paclitaxel nanoformulations (Abraxane^®^). However, the clinical benefits of such therapies are not yet clear, and several challenges remain in translating them into clinical practice (29). Recently, significant progress has been made in the field of cancer nanotherapy, particularly in the exploration of magnetic nanoplatforms for the treatment of NSCLC (29). In this study, the focus will mainly be on the application of nanoparticles in the immunotherapy treatment of NSCLC, taking into account their potential for anti-tumor and anti-progression effects, as well as the potential of nanomedicine-based technology. Furthermore, various translational studies will be explored and discussed regarding the utilization of nanomedicine-based technology and biomarkers in NSCLC immunotherapy.

## Mechanisms of nanoparticle therapeutics

**Figure 1:**
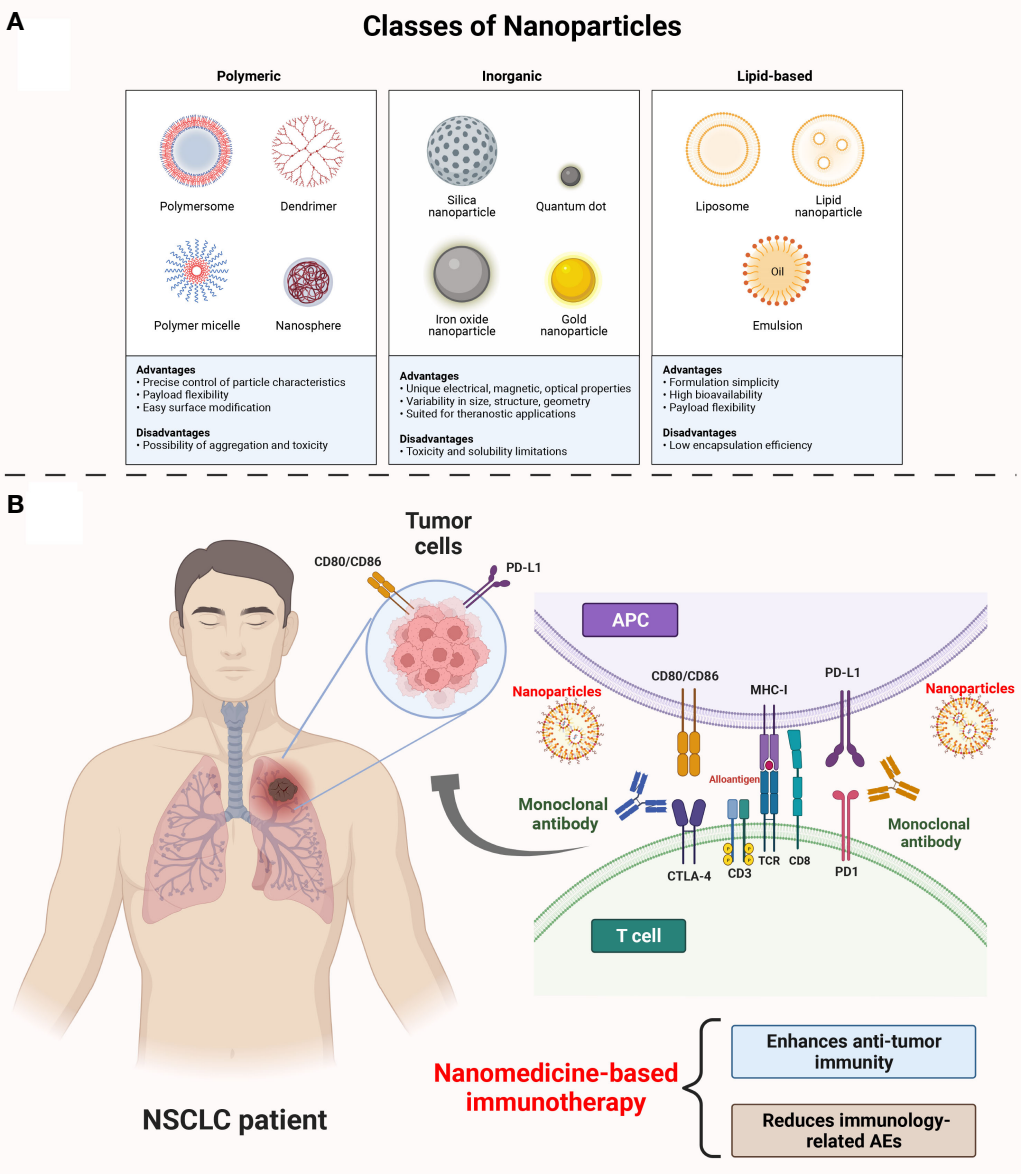
The mechanisms of nanoparticles medicine in NSCLC immunotherapy. **(A)** Classes of nanoparticles. Mainly, there are three classes of nanoparticles: polymeric, inorganic, lipid-based nanoparticles. The polymeric nanoparticles are precise control of particle characteristics, payload flexibility and with easy surface modification. The inorganic nanoparticles are unique, electrical, magnetic, optical properties. They can be visualized in size, structure, geometry and they are suited for theranostic applications. The lipid-based nanoparticles are of formulation simplicity, high bioactivity and payload flexibility. **(B)** Nanoparticles loading ICIs monoclonal antibody in NSCLC treatment. Administration of ICIs leads to the activation of tumor-specific T cells (always CD8+ T cells) activation by blocking PD-1 and CTLA-4 on T cells, and PD-L1 and CD80/86 on APC/tumor cells as well. The use of ICIs along will over-stimulate the immune system and increase the potential of off-target effects which is the source of systematic AEs. Specially, nanoparticles loading ICIs can lead to a high local concentration of ICIs on the tumor region while reducing the off-target effects from ICIs. NSCLC, non-small cell lung cancer; ICIs, immune checkpoint inhibitors; PD-1, programmed cell death 1; CTLA-4, cytotoxic T-lymphocyte–associated antigen; PD-L1, programmed cell death ligand 1; APC, antigen processing cell; AEs, adverse events.

There is considerable evidence that checkpoint blockade drugs, which primarily inhibit adaptive immunity, are effective and have manageable side effects, including intestinal and pulmonary toxicity and autoimmune sequelae in patients (29). Although new immunotherapeutic approaches aimed at enhancing adaptive responses through immunostimulatory pathways have been met with many concerns regarding their safety profile, these concerns have hindered their successful implementation (29). Nanomedicine offers a promising solution to overcome the limitations of conventional drug delivery methods and maintain the ability to target specific tissues or cell types. It involves the formulation of drugs within carrier materials that are smaller than 100 nanometers. By using nanocarriers, many immunotherapies can be made safer and more effective. The conversion of immunotherapy into nanoparticles comprising of lipids, polymers, or other materials has resulted in changes in systemic exposure, facilitated tumor accumulation, improved innate immune compartments, and modified single-cell signaling (**Figure 1**). As a result of such approaches, drug development becomes more complex, but these technical difficulties can be easily overcome by numerous clinical-stage companies (30–32). The original aim of using nanomedicine-based drug formulations was to improve the pharmacokinetics and safety profiles of chemotherapy, as well as to increase the accumulation of drugs within tumors (**Table 1**). The potential for improved efficacy of cancer immunotherapy can be attributed to the ability of nanomaterials to accumulate immune-drugs within cancer cells or around the tumor microenvironment (TME). Furthermore, nanomaterials offer new mechanisms of action for immunotherapeutic agents, including the ability to display ligands on immune cells, modulate the delivery of cell-impermeable compounds intracellularly, and adjust the pattern and timing of drug release or activation.

**Table 1:** Currently available nanomedicine-based chemotherapeutic drugs in clinical trials.

Drug	Nano delivery system	NSCLC stage	Phase	Registration number
Doxorubicin	pegylated liposome	IIIB–IV	II	NCT01051362
Hydrochloride	aerosolized liposome	IIIB	I	NCT00020124
(Adryamycin^®^, Rubex ^®^)	liposome	IIIB–IV	IV	NCT02996214
Paclitaxel	Polymeric micelle (Genexol-PM^®^)	IV	II	NCT01770795
Camptothecin	aerosolized liposome	IIIB–IV	pre-clinical	NCT00277082
Lurtotecan	liposome	IIIB	I	NCT00006036
Paclitaxel and carboplatin	ABRAXANE^®^	IV	III	NCT03875092, NCT02775435
Abraxane, cisplatin and carboplatin	ABRAXANE^®^	III	II	NCT02662634
Paclitaxel, cisplatin and carboplatin	ABRAXANE^®^	IIB, IIIA	II	NCT02016209
Docetaxel	BIND-014	III	II	NCT01792479
Docetaxel	BIND-014	III (KRAS-positive)	II	NCT02283320
Paclitaxel and carboplatin	ABRAXANE^®^	III	III	NCT04033354
Paclitaxel and carboplatin	ABRAXANE^®^	III	II	NCT00553462
ABI-007	ABRAXANE^®^	I/II	IV	NCT00073723

## Nanoparticles for immunogenic tumor cell death promotion

In the case of immunogenic cell death (ICD), ATP and high mobility group protein B1 (HMHB1) known as extracellular molecular patterns are released, along with calreticulin and heat shock protein 90 (HSP90) being exposed to the surface (33). These factors contribute to the recognition of tumor antigens by antigen-presenting cells and their subsequent activation. Nanomedicine formulations can be used to promote ICD because they help to accumulate cytotoxic agents within tumor cells.

Furthermore, the direct association between nanomaterials and external energy sources permits the up-regulation of ICD in response to radiotherapy and magnetic hyperthermia therapy etc (33). The utilization of nanoparticle-based formulations of chemotherapeutic agents that promote immunogenic cell death (ICD) can augment anti-tumor immunity by improving drug delivery to tumor cells. In comparison to free doxorubicin, doxil treatment resulted in increased intratumoral CD8+ T cell infiltration, reduced regulatory T cells (Tregs), and increased CD80 expression by myeloid cells. Currently, the combination of doxil and checkpoint blockade is being investigated in metastatic breast cancer patients. Moreover, a novel approach to improve doxorubicin delivery and enhance ICD involves the covalent linkage of synthetic high-density lipoprotein (HDL) nanodiscs to nanoparticles using an acid-labile linkage (33).

The use of radiation can be regarded as a component of combination immunotherapies (33). Although radiation therapy can cause irreversible DNA damage to cancer cells, it can also trigger the cyclic AMP synthase stimulator of interferon genes (cGAS-STING) pathway, which generates pro-inflammatory cytokines and thereby induces innate and adaptive anti-tumor immunity (33). A high radiation dose could result in attenuated STING activation due to DNA exonuclease TERX1-dependent degradation of cytosolic DNA (33). The immunosuppressive immune cells attacking the tumor can also blunt an initial successful pro-inflammatory radiotherapy (33). In some preclinical studies, radiotherapy has been shown to activate tumor-resident T cells, which have a greater resistance to radiotherapy than circulating T cells (33). The intratumoral administration of nanoparticles post-radiotherapy facilitated the identification of protein antigens that were released from the cancer cells undergoing cell death induced by the nanoparticles. Subsequently, these protein antigens were transported through the lymphatic vessels and were taken up by phagocytic professional antigen-presenting cells located in the draining lymph node (33). By engaging directly with the ICD, radioenhancers can likewise be employed as nanomedicines to improve the effects of ionising radiation (33). Utilizing externally applied alternating magnetic fields to activate paramagnetic iron oxide nanoparticles within the tumor microenvironment (TME), localized hyperthermia can be generated. Preclinical models of glioma, colon cancer, and melanoma have demonstrated the effectiveness of this approach (34, 35), this method encourages CD8+ T cell-mediated immunological responses. Nanoparticle-mediated hyperthermia can induce the production of various pro-inflammatory cytokines within the treated tumor and activated dendritic cells (DCs). In a preclinical study using a dog model, an oral melanoma growing alone was successfully treated with radiation, a virus-like particle adjuvant, and magnetic nanoparticle-induced hyperthermia (36). Numerous nanomaterials have also been employed to increase the effectiveness of combination therapies, either by integrating drugs with synergistic modes of action to produce supra-additive effects, or by encapsulating multiple medications in a single particle to ensure co-delivery to target cells (37, 38). Nanoparticles were prepared using a phospholipid shell containing dihydroartemisinin, a second drug that produces reactive oxygen species (ROS). In preclinical models of colorectal adenocarcinoma, this combination therapy-loaded nanoparticle significantly increased the incidence of ICD in tumors and showed synergistic effects with anti-PD-L1 therapy (39). Phase I clinical trials have just begun for these nanoscale coordination polymer formulations that contain unidentified TME modulating compounds. Additionally, nanoparticles may be made to interact with outside energy sources and carry immunostimulatory agents. When combined with infrared light irradiation, inorganic nanoparticles coated with lipid-anchored photosensitizers enhanced calreticulin exposure on tumor cells and immune cell infiltration in mouse breast tumors (39). In murine models of colon cancer, a single nanoparticle was formulated to encapsulate oxaliplatin, which enabled the combination of photodynamic therapy and chemotherapy to induce ICD, leading to the regression of both irradiated primary tumors and unirradiated secondary tumors (39).

## Immunoregulatory receptor engagements

Additionally, T cells and natural killer cells receive costimulatory signals. Many important immunoregulatory receptor interactions occur at cell-cell interfaces. By presenting ligands on the surface of particles at physiologically relevant sizes, nanomedicine offers the potential to mimic such an interface. Compared to equivalent concentrations of soluble anti-CD137 antibodies, agonistic anti-CD137 antibodies conjugated to liposome surfaces were ten times more effective at activating CD8+ T cells (39). Due to the limited capacity of each individual nanoparticle to emit only one signal, as well as its relatively small size which is insufficient to elicit significant receptor cross-linking, the activation of T cells is suboptimal when magnetic nanoparticles of 30 nm diameter, containing immobilized peptide MHC molecules, are coupled with anti-CD28 antibody nanoparticles (39). Activation, proliferation, and the induction of effector functions in T cells are all strongly enhanced when a magnetic field is employed to cause nanoparticle clustering. Additionally, particles can provide several ligands to concurrently target different cell types or bind multiple receptors on target immune cells. Nanoparticles comprising anti-programmed cell death 1 (anti-PD-1) as well as anti-OX40 antibodies improved T-cell activation and therapeutic effectiveness in mice with melanoma and breast cancer when compared to therapy with the same drugs given as a straightforward medication combination (39).

Nanoparticles have been demonstrated to present clusters of death-inducing ligands to circulating tumor cells *via* the surface of immune cells. Liposomes conjugated with recombinant E-selectin and tumor necrosis factor-related apoptosis-inducing ligand (TRAIL) on their surface enable T cells, monocytes, and natural killer cells to attach to them, allowing for the delivery of clustered TRAIL to circulating tumor cells. When these death ligands are activated, tumor cells are killed (39). TRAIL-presenting nanoparticles have been demonstrated to prevent tumor metastasis and prolong survival in both murine and human xenograft tumor models (40, 41). Lipid nanoparticles have been employed to transport a small molecule inhibitor of macrophage colony-stimulating factor 1 receptor (CSF1R) signaling, in conjunction with signal-regulatory protein-α (SIRPα) blocking antibodies, for the purpose of reprogramming tumor-associated macrophages (TAMs) from an immunosuppressive state to a tumoricidal state in both murine and human xenograft tumor models (42). This study highlights the enhanced effectiveness of the antibody, which can be attributed to its dual role as both a targeting molecule and a direct therapeutic agent by inhibiting SIRPα signaling. Thus, nanomedicine formulations have the potential to modify various immunotherapies targeting cell surface receptors’ signaling to augment their anti-cancer efficacy.

## Signals associated with cytosolic drug delivery

Nanomaterials have been widely employed in medicine to facilitate the direct delivery of drugs into the cytosol. However, hydrophilic and charged substances like nucleic acids, which are taken up by cells *via* endocytosis, often get trapped in the endolysosomal pathway. As a substitute for natural viruses, nanomaterials have been extensively studied to facilitate the entry of nucleic acids and other drugs into the cytosol. The delivery of substances that activate cytosolic danger sensor proteins, RNA, or DNA encoding immunomodulatory proteins is crucial in the context of cancer immunotherapy. Recently, it has been demonstrated that cyclic dinucleotide agonists of STING may be delivered to the cytoplasm using polymersomes (42). Upon intracellular uptake, polymersomes undergo endosomal acidification, leading to the rupture of endosomal membranes and the release of their STING agonist payload into the cytoplasm. The intratumoral administration of these nanomaterials increased the potency of cyclic dinucleotides by more than 100-fold and demonstrated increased anti-malarial efficacy in melanoma models. Furthermore, a liposomal formulation of cyclic dinucleotides containing pH-responsive lipids was shown to enhance STING activation (42). Alternative approaches to using nanomaterials to activate danger signal pathways involve using synthetic polymers. One recent study has demonstrated the use of pH-responsive block copolymer micelles that can rupture endosomes and directly engage with STING to activate downstream signaling (42). Furthermore, the functionalization of nanoparticles with immunostimulatory agents, such as toll-like receptors (TLRs) agonists, has been shown to be effective. These immunostimulants are absorbed more efficiently by myeloid and antigen-presenting cells compared to free TLR ligands, resulting in heightened immunostimulation (43, 44).

Further control of immunostimulatory or immunosuppressive pathways may be possible through delivery of nucleic acid cargoes encoding therapeutic proteins (45). Oxaliplatin chemotherapy combined with systemic injection of a lipid- and protamine-based nanoparticle expressing a PD-L1 trap protein reduced tumor development in a metastatic colon cancer model (45). Intratumoral administration of lipid nanoparticles that express mRNA for the cytokines IL-23 and IL-36γ, along with the T-cell costimulator OX40L, have led to *in situ* vaccination and CD8+ T-cell dependent tumor regression in both colon cancer and melanoma models. This approach is currently being investigated in clinical trials (45). Furthermore, the delivery of anti-cancer therapy through surface-engineered nanoparticles targeting tumor cells has the potential to mitigate unexpected effects on off-site targets, while increasing the therapeutic concentration at the site of action, efficacy, and safety for treating lung cancer. By co-delivering therapeutic agents at the optimal time and location using smart nanotools, a simultaneous effect on multiple signaling pathways can be achieved, thus avoiding or combating resistance and preventing undesirable effects. This is the underlying theoretical basis for using designed nanoparticles (46–48). Preclinical and clinical trials have demonstrated significantly improved progression-free survival (PFS) in patients with epidermal growth factor receptor (EGFR)-mutant non-small cell lung cancer (NSCLC) who receive a combination of EGFR tyrosine kinase inhibitors (EGFR-TKIs) and angiogenic therapy. Nonetheless, the use of this combination therapy has been associated with an elevated incidence of grade 3-5 adverse reactions (49–53). Nanomedicine strategies can be utilized to enhance the targeted delivery of combination therapy to the site of action, leading to improved treatment outcomes and reduced incidence of adverse events. Innovative nanomedicine approaches offer the potential to interact with tumor cells through diverse mechanisms, thereby overcoming the limitations associated with conventional antibody-drug conjugates (54, 55). Molecular targeting has also shown great potential in cancer treatment, and their combinations with conventional chemotherapy have improved PFS in a phase III clinical trial with EGFR-mutant NSCLC patients (56).

Nanomedicine has the potential to address the limitations associated with co-delivery of combined therapies by providing a targeted delivery approach for high concentrations of anticancer drugs at the site of action. After absorption from the gastrointestinal tract and distribution throughout the body, EGFR-TKIs interact with the EGFR signaling pathways of many normal cells, affecting proliferation, differentiation, migration, and apoptosis. The use of nanomedicines for targeted drug delivery could help resolve these issues by (1) improving the pharmacokinetic profile of the drug, (2) enhancing tumor targeting potential and localization at the tumor site, (3) minimizing off-site targets and side effects, (4) reducing or reversing multidrug resistance mechanisms, and (5) inhibiting acquired resistance and sensitizing tumor cells to EGFR-TKIs through synergistic action against various anti-tumor targets (57, 58). In addition, the use of nanotechnology as a tool for targeted delivery may offer improvements in the efficacy of anticancer drugs and help to identify beneficial synergistic combinations for treating different subtypes of lung cancer. Nanomedicines can facilitate (1) multivalent targeting and co-delivery of agents to endothelial cells, tumor cells, and the tumor microenvironment; (2) the delivery of large payloads of active substances with diverse physicochemical properties; and (3) the limitation of resistance mechanisms (59). Nanotherapy has the potential to revolutionize the field of clinical lung cancer treatment by reducing the risk of therapeutic failure caused by non-coordinated co-delivery of therapeutic agents and off-target side effects. Despite significant advancements, precise control over the *in vivo* trajectories of nanosystems is yet to be achieved.

## The control of immunostimulation

Immunotherapy dosing schedules can have a significant impact on treatment efficacy in preclinical models. Nanoparticles have long been employed to slow down the fast clearance of medicines that might otherwise enter tissues or the bloodstream, and they may have a similar function in immunotherapy (45). To accurately manage the timing of medication release, a nanomedicine formulation can also be made to interact with outside energy sources like heat or light. A TLR9 agonist called CpG-containing DNA complexed with near-infrared light-activatable nanoparticles has been used by researchers to establish the viability of this strategy (45). By controlling the time and place of immunostimulation, as well as the signalling of immunostimulatory biologics, nanotechnology enables more accurate use of current immunotherapy techniques.

## Nanomedicine-based immunotherapy for NSCLC

The cornerstones of NSCLC treatment in recent times have been surgery, chemotherapy, as well as radiation, but their efficiency has gradually decreased over time and their adverse effects have compelled study into other strategies. Overall survival rates for NSCLC patients have plateaued with the advent of molecularly targeted treatments in conjunction with chemotherapy (60–62). The next generation of medications that will enhance patients’ overall response to NSCLC treatment should be developed as a result of using immunotherapy in conjunction with gene therapy.

## Immunotherapy

As evidenced by the growing number of clinical trials (63, 64), immunotherapies have recently become more focused (**Table 2**). There are several ways in which the immune system is stimulated by these treatments, and these treatments vary according to the patient’s genetic and epigenetic alterations. Based on histological findings, the immune system was assumed to have little or no capacity to respond to tumors. However, the idea of immunosurveillance demonstrates that a tumor may be identified and addressed at an early stage of development by both the innate and adaptive immune systems (65, 66). Tumors continue to develop defence mechanisms, an immunosuppressive TME, and ways to elude the actions of natural killer (NK) cells, CD8+ T cells, CD4+ T cells, and macrophages despite immunosurveillance. The hypothesis of immunoediting, which has three stages—elimination, equilibrium, and escape—can be used to explain how the immune system and the tumor communicate (66). Immunotherapy’s primary objective is to make tumor-infiltrating immune cells more sensitive to the elimination process. A number of approaches are being investigated to achieve this goal, including the design of vaccines, the modification of immune cells, and the inhibition of mechanisms that allow tumors to evade detection.

**Table 2:** Key clinical trials investigating efficacy and safety of nanomaterials for lung cancer immunotherapy.

Trial id	Cancer details	Status	Location	Duration	Drug	Delivery system	Institute/Agency
NCT00291473	all stage NSCLC and other carcinomas	phase I	Japan	2005-2008	Mixed cancer vaccines, CHP-HER2 and CHP-NY-ESO-1 with OK-432 (picibanil)	cholesterol-bearing hydrophobized pullulan	Ludwig Institute for Cancer Research
NCT00157209	stage IIIB/IV NSCLC	phase IIB	Germany	2000-2012	Tecemotide with single-dose low CPA	liposome	Merck KGaA, Darmstadt, Germany
NCT00157196	stage IIIA NSCLC	phase II	the USA	2005-2012	Tecemotide with single-dose low CPA	liposome	Merck KGaA, Darmstadt, Germany
NCT00409188	unresetable stage III NSCLC	phase III	23 countries	2007-2012	Tecemotide with single-dose low CPA	liposome	EMD Serono
NCT00960115	unresetable stage III NSCLC	phase I/II	Japan	2009-2015	Tecemotide following chemotherapy	liposome	Merck KGaA, Darmstadt, Germany
NCT01015443	unresetable stage III NSCLC	phase III	Asia	2009-2015	Tecemotide following primary chemo-radiotherapy	liposome	Merck KGaA, Darmstadt, Germany
NCT02049151	unresetable stage III NSCLC	phase III	the USA	2014-2015	Tecemotide following concurrent chemo-radiotherapy	liposome	EMD Serono/Oncothyreon Canada Inc
NCT01853878	all stage NSCLC	phase II	9 countries	2013-2016	Recombinant PRAME protein combined with the AS15 adjuvant system GSK2302032A	liposome	GlaxoSmithKline
NCT01258868	all stage NSCLC and other carcinomas	phase I	the USA	2013-2016	Tumor Cell vaccines with ISCOMATRIX adjuvant and celecoxib	liposome	National Cancer Institute (NCI)
NCT00828009	unresetable stage III NSCLC	phase II	the USA	2010-2019	Tecemotide with bevacizumab after chemotherapy and radiation therapy	liposome	ECOG-ACRIN Cancer Research Group
NCT03836352	all stage NSCLC and other carcinomas	phase II	the USA and Canada	2018-2023	DPX-Survivac with low dose cyclophosphamide & pembrolizumab	liposome	ImmunoVaccine Technologies, Inc. (IMVInc.)

In some preclinical studies, nanoplatforms have been developed for cancer immunotherapy. Moon and his colleagues created a stable and uniform lipoprotein nanodisc, which included phospholipids and apolipoprotein mimetic peptides consisting of 22 amino acids, for delivering neoantigen vaccines to the lymph nodes that drain tumors. These nanodiscs stimulated a potent T cell immune response against tumors, which led to the eradication of established tumors and inhibited the growth of metastatic tumors in murine lungs (67). Nanotechnology and nanomaterials have been utilized to enhance molecularly targeted immunomodulation. This is because molecular targeting drugs have been found to initiate immune responses through various mechanisms, such as aiding in antigen presentation by antigen-presenting cells (APC), promoting T cell infiltration in the tumor region, triggering natural killer (NK) cells, instigating immunogenic cell death (ICD) in tumor cells, and reducing the number of myeloid-derived suppressor cells (MDSCs), regulatory T cells (Treg), and tumor-associated macrophages (TAMs) in the tumor region (68). The A549 tumor xenograft experiment demonstrated a significant antitumor effect, concomitant with the robust induction of innate and adaptive immune responses. The infiltration levels of both CD8+ and CD4+ T cells were augmented, and NK cells were activated, concomitant with the reduction of myeloid-derived suppressor cells (MDSCs) and regulatory T cells expressing the Foxp3 transcription factor (Foxp3+ Tregs), known for their role in immune tolerance (69). Numerous studies are still underway to investigate the potential of immunomodulatory nanomedicine in lung cancer immunotherapy, and we anticipate a surge in their applications in the near future (70–72).

## Inhibitors of immune checkpoints

The currently favored treatment modalities being employed are immune checkpoint inhibitors (ICIs) (61, 73). These medications target the immune system’s control mechanisms for lymphocyte activation. The immune checkpoint mechanisms CTLA-4 and PD-1 have undergone substantial research and analysis. T cell activation is inhibited by both mechanisms in a different manner and at different levels (74). By utilizing CTLA-4-dependent mechanisms, the immune system is prevented from overreacting in the early stages of activation. Antigen presentation causes T lymphocytes to become activated, and this is when CTLA-4 is expressed on the cell surface. The receptor engages in interaction with CD80 and CD86, which are expressed on antigen-presenting cells’ membranes (APCs). Through the production of numerous cytokines as a result of this interaction, the TME inhibits and boosts the immune response. Antibodies having a high recognition specificity for CTLA-4 are given as part of a treatment with CTLA-4 inhibitors to stop the protein from binding to CD80/86 ligands[68]. On the other hand, during the effector phase of the innate specific immune response, the PD-1/PD-L1 pathway downregulates the immune response in the late stages. PD-1 is expressed on the membrane of T/B cells and natural killer cells as a result of its activation. In healthy systems, the interaction of this protein with PD-L1 and PD-L2 ligands can lower T cell activation and hence avoid an autoimmune response. But in the TME, tumor cells either produce these ligands on their surfaces or enable other immune cells to express them by activating molecules like IFN-γ. The TME can restrict T cell activation, proliferation, survival, and effector activities in addition to dampening the immune response. For instance, the introduction of anti-PD-1 antibodies blocks the protein’s interaction with PD-L1 and PD-L2, blocking the pathway’s downregulation. The PD-L1 protein is sequestered, which also prevents T-cell activation, and thus prevents the effects of other medications. Due to the possibility of PD-1 engagement with PD-L2, the downregulation of the pathway is limited; thus, the former strategy reduces the immune-related adverse events associated with this therapy. The predominant form of administration for an inhibitor of the PD-1/PD-L1 and CTLA-4 pathways is immunoglobulin G. (IgG). After rigorous clinical studies, several ICIs have been licensed for the treatment of NSCLC (75, 76). ICIs are useful for individuals with advanced cancer or as a second-line therapy, according to clinical trials. Pembrolizumab and atezolizumab have both been administered alone, however chemotherapy is the setting in which they are most frequently utilized (46, 77–79). Clinical data to date reveal that ICIs only assist a small percentage of patients and have limited response rates, despite their powerful antitumor properties (80). Recent research has demonstrated that the administration of these compounds along with chemotherapeutic drugs that are known to influence immune function increases the anti-tumor activity of ICIs. Preclinical investigations have indicated that the amalgamation of ICIs with nanoparticle-mediated chemotherapy results in favorable outcomes in mouse tumor models. A former study described the formulation and development of a drug delivery system comprised of high-density synthetic lipoprotein (sHDL) nanodiscs for chemotherapeutic agents. Through the implementation of this type of platform for delivery, various chemotherapy drugs can be safely and effectively released, and the immune response can be enhanced by inhibiting the PD-1/PD-L1 pathway (67, 81).

ICIs can be used in conjunction with drug-loaded nanoparticles in addition to co-administration with chemotherapy (82). One of the best methods now available is nanoparticle-assisted photodynamic and thermal therapy (82). Photothermal therapy (PTT) is a minimally invasive approach that utilizes nanomaterials to release vibrational energy and ablate cancer cells in localized malignancies. PTT is an alternative to checkpoint inhibitors and has the advantage of allowing tumors to overcome their adaptive immune evasion mechanisms through combined therapy (82). The strategy involves the use of iron oxide (Fe3O4 superparticles) along with magnetic resonance imaging (MRI) particles that have already been approved by the FDA, contained within spheres of the copolymer mPEG-poly(lactide-co-glycolide) (PLGA). Additionally, the immune adjuvant Toll-like receptor 7 (TLR7) is encapsulated with the magnetic nanoparticles to promote an organised immune response against tumors. The effectiveness of this approach has been demonstrated through direct tumor elimination during NIR irradiation, and activation of dendritic cells, by combining three FDA-approved components with PD-L1 ICIs (83).

## Tumor vaccines

In recent years, several types of vaccines have been developed into therapeutic vaccines (83). By enhancing humoral and cellular T-cell immune responses, these vaccines were then developed to cure a disease. Through the detection of altered proteins that are expressed in an abnormal manner by tumor cells, the concept of an anti-cancer vaccine was developed. These treat disease by enhancing the humoral and cellular responses of the immune system, primarily T cells. Tumor vaccines are developed by identifying mutated proteins that are abnormally expressed by tumor cells. The immune system identifies these as tumor-associated antigens (TAAs) and categorizes them as expressed fetal antigens (FAs), and further categorizes them as expression of detail antigens (always absent in healthy adults) and overexpression of normal proteins (84–86). The idea behind therapeutic vaccinations is to train the immune system to recognise and react to specific antigens. For the therapy of NSCLC, many vaccine techniques have been examined. These comprise whole-cell vaccines (87–89), protein- and peptide-based vaccines (90, 91), comprise mRNA vaccines (92, 93) and vaccinations based on the whole cells (**Table 3**).

**Table 3:** Currently available clinical trials evaluating cancer vaccines for NSCLC.

Vaccine	Components	NSCLC stage	Clinical trial phase	Registration number
**Cellular vaccine**	Allogenic tumoral cells (1650-G)	I–II	II	NCT00654030, NCT00601796
	Autologous engineered dendritic cells (MIDRIX4-LUNG)	III	I	NCT04082182
	Autologous mRNA/DNA transfected dendritic cells (MIDRIXNEO-LUNG)	III–IV	I	NCT04078269
	Allogenic mRNA-transfected dendritic cells (AST-VAC2)	III–IV	I	NCT03371485
	Allogenic engineered dendritic cells irradiated III–IV I NCT03371485 with seven active agents (NY-ESO-1, MAGE C1, 4MAGE C2, TPGB, Survivn, MUC1, Melan-A antigen (PDC*lung01)	NS	I-II	NCT03970746
	Autologous dendritic cells pulsed with allogenic tumor cells	III	II	NCT00103116
	Allogenic whole tumor cells (Lucanix ^®^)	III–IV	III	NCT00676507, NCT01058785
	Autologous dendritic cells pulsed with allogenic tumor cells (MelCancerVac^®^)	III–IV	II	NCT00442754
	Autologous dendritic cells pulsed with p53 peptide	III	II	NCT00019929
	Engineered autologous killed tumor cells	IV	I–II	NCT01159288, NCT02439450
	Allogeneic CD4+ memory Th1-like T-cells (Allostim^®^)	II–IV	I–II	NCT01065441
	Autologous dendritic cells pulsed with allogenic tumor cells (DVAC/LuCa)	IV	I–II	NCT02470468
	Allogenic lymphocytes	I–IV	I	NCT00161187
	GVAX with autologous tumor cells mixed with an allogeneic GM-CSF-secreting cell line	IV	I-II	NCT00074295
	Belagenpumatucel-L with 4 TGF-β2-antisense gene-modified, irradiated, allogeneic NSCLC cell lines	III-IV	III	NCT00676507
**Protein vaccine**	MUC1	III	I–II	NCT01720836, NCT03353675, NCT03623750
	Heat shock protein (gp96-Ig)	III–IV	I	NCT00503568
	Tumor antigen-loaded dendritic cell-derived exosomes	III–IV	II	NCT01159288
	Anti-idiotype vaccine	IIA–III	II	NCT00006470
	Recombinant PRAME protein	I–IIIA	II	NCT01853878
**Peptide vaccine**	IDO peptide	III–IV	I	NCT01219348
	HLA-A*0201 restricted 9-mer epitopes (Vx001)	IV	II	NCT01935154
	Short lived proteins (SLiPs) and defective ribosomal products (DRiPs)	III–IV	I	NCT00850785, NCT01909752
	Synthetic peptides encoding hTERT (UV1)	III	I–II	NCT01789099
	MUC1 peptide (Tecemotide/L-BLP25/Stimuvax^®^)	III	III	NCT00409188, NCT00960115, NCT00157196, NCT00828009, NCT00157209
	UCP2 and UCP4 (telomerase derived peptides)	III	I–II	NCT02818426
	Epitope Peptide Restricted to HLA-A*02	III–IV	I	NCT01069640, NCT01069575
	GV1001 (Synthetic peptides encoding hTERT)	III	N.E. (already approved in Korea for pancreatic cancer)	NCT00509457
	(MAGE3 epitope) (Astuprotimut-R (GSK-249553))	IB–II	II	NCT00290355
	Wilms tumor 1 (WT1) analog peptide (DSP-7888)	III–IV	I	NCT03715985
	Peptides derived from a patient’s tumor individual neo-antigens (NeoPepVac, GRT-C901 and GRT-R902, GEN-009, NEO-PV-01)	III–IV	I	NCT03715985, NCT03639714, NCT03794128, NCT03953235, NCT03633110, NCT02897765, NCT03380871
	Tedopi^®^ (OSE2101)	III–IV	III	NCT02654587
	RAS peptide	II–IV	I–II	NCT00019006, NCT00019331, NCT00003125
	Arginase-1 peptide	Generic	I	NCT03689192
	YE-NEO-001 Neoepitope yeast vaccine (YE-NEO-001)	Generic	I	NCT03552718
	MAGE-12 peptide	IV	I	NCT00020267
	Patient specific neoepitopes	IV	I	NCT00020267
	CIMAvax-EGF	III-IV	III	–
	Racotumomab-alum based on NeuGcGM3	III-IV	III	NCT01460472
	PRAME based on PRAME	IB-IIIA	I	NCT01159964
	MAGE-A3	IB-IIA	III	NCT00480025
**mRNA vaccine**	NY-ESO-1, MAGE C1, 4MAGE C2, TPGB, Survivn, MUC1 (RNActive^®^)	III–IV	I–II	NCT03164772, NCT00923312
	KRAS gene vaccine V941 (mRNA-5671)	III–IV	I	NCT03948763
	Personalized vaccine against patient’s mutations (RO7198457)	III–IV	I	NCT03289962
	BI 1361849 mRNA vaccine	III-IV	I/II	NCT03164772
**DNA vaccine**	NY-ESO-1 plasmid DNA (pPJV7611) to increase immunogenicity of tumor cells	III–IV	I–II	NCT00199849
	Plasmid encoding neoepitopes (VB10.NEO)	III–IV	I–II	NCT03548467
**Virus/vector**	TG4010 based on MUC1	III–IV	II	NCT00415818
	LV305 based on NY-ESO-1	III–IV	I	NCT02122861
	Ad-MAGEA3 with MG1-MAGEA3 (adenovirus vector maraba virus)	III–IV	I/II	NCT02879760
	CVA21 (coxsackie virus)	III–IV	I	NCT02043665
	VSV-IFNβ-NIS (vesicular stomatitis virus)	III–IV	I/II	NCT03647163
	REOLYSIN (reovirus serotype 3—dearing strain)	III–IV	II	NCT00861627, NCT01708993
	(NTX-010) Seneca Valley virus-001 (seneca virus, small cell lung cancer)	III–IV	II	NCT01017601
	rAd-p53 (adenovirus)	III–IV	II	NCT01574729
	ADV/HSV-tk (herpes simplex virus)	III–IV	II	NCT03004183, NCT02831933

## Vaccines that are based on proteins and peptides

Along with whole tumor cell vaccinations, one of the earliest and most promising approaches to treating cancer is the use of proteins or peptides. However, peptide vaccines are limited by: (1) the low immunogenicity of cancer antigens alone, which requires the co-administration of an adjuvant to stimulate the immune response (84, 85). (2) Additionally, the possibility of an autoimmune reaction being set off is increased by the absence of proteins that are uniquely expressed in cancer cells. Antigenic proteins contain complicated glycosylation patterns and are challenging to purify, in addition to safety issues. The bench to bedside approach is hampered by this. Utilizing peptides enhances stability and selectivity while lowering the negative immunological reactions brought on by using full proteins. However, it has been demonstrated that a variety of protein vaccines are efficient therapies for NSCLC. A3 (MAGE-A3) is a melanoma-associated antigen that is nearly exclusively expressed on various types of tumor cells. Although these outcomes are not therapeutically applicable, MAGE-A3 treatment in conjunction with immune response-enhancing adjuvants has been shown to provide favorable results (94). Mucinous glycoprotein-1 (MUC1), another well-studied protein linked with tumors, is used as an antigen in the TG4010 vaccination. On the other hand, the use of a 25-amino-acid MUC1 peptide has demonstrated outstanding outcomes (95, 96), but a vaccination based on the whole MUC1 protein has failed to obtain substantial favorable results in numerous clinical trials (94). The issue of peptide stabilization and preservation can be resolved by enclosing peptides within lipid particles, which can also enhance their uptake by antigen-presenting cells (APCs). Furthermore, these vehicles can be decorated with immunopotentiators such as immune cell-targeting adjuvants or ligands (97).

## mRNA vaccines

As a result of testing the expression of proteins from injected mRNAs, mRNA vaccines appeared in the early 1990s (97). Initial research on vaccines containing genetic material had mainly focused on DNA. This could be attributed to the higher stability of DNA when compared to RNA. However, the development of drug delivery nanosystems and the safety of mRNA regarding mutagenicity and internalization accessibility have shifted the focus towards mRNA (97, 98). Transfecting cells with mRNA can trigger the immune response in various ways. One of them is by training antigen-presenting cells (APCs) to recognise the encoded antigens and activate humoral and cellular immune responses through the use of mRNA that encodes one or more tumor-associated antigens (99). Due to the drug’s outstanding effectiveness and safety profile, more clinical trials are now being conducted. One of the most promising vaccines now on the market is RNActive^®^ CV9201. It is made up of a combination of five NSCLC-associated antigens that prompt an immune response either after dendritic cells have been collected, transfected, and given to the patient, or after the patient has received mRNA directly (99). Over 65% of patients with NSCLC were able to establish an immunological response to the vaccination, according to findings from a phase Ib clinical study of an mRNA vaccine. Forty-eighty percent of patients had distinct antigen-specific humoral responses, despite the fact that the degree of the reaction elicited varied (99). The mechanism of action of the CV9201 vaccine involves the formation of a complex between mRNA and protamine, which is a cationic protein that can bind to harmful substances and facilitate their internalisation by cells. *In vivo* studies using this protein-based nanoparticle have shown promising results, inducing activation of the adaptive immune response (99). In spite of this, it is worth mentioning that, since it is autologous, its application to the healthcare system is not affordable. In other studies, EVs such as exosomes were found to be the most promising in the development of cancer vaccines (99). As a result of these vesicles, both mRNA-based antigens can be released directly into the body to induce an immune response, as well as embryonic cells can be co-cultured with antigen-loaded exosomes *in vitro* in order to mature and then be injected to patients (99). Additionally, to increase the immunogenicity of vaccines, these vesicles are being created *via* membrane decorating with viral fusion proteins or Toll-like receptor ligands (99).

## liposomal vaccine

A liposomal cancer vaccine (L-BLP25) was established by Oncothyreon Canada Inc., where the antigen tecemotide (carcinoma-associated human MUC-1) and an adjuvant 3-O-Deacyl-4’-monophosphoryl lipid A (MPL) were integrated into the lipid bilayer made up of 1,2-dipalmitoyl-sn-glycero-3-phosphocholine (DPPC), 1,2-dimyristoyl-sn-glycero-3-phospho-(1’-rac-glycerol) (DMPG), and cholesterol. Among many global clinical trials, a phase IIb trial (NCT00157209) with patients with patients with stage IIIB or IV NSCLC demonstrated an increment of 4.2 months in median survival in the L-BLP25-administration group compared to the cohort receiving best supportive care only. In a subcluster comprising patients with stage IIIB loco-regional NSCLC, an enhancement of 17.3 months in median survival was observed, where the treatment group showed 49% 3-year survival rate over 27% of best supportive care group (100).

More light should be shed on the tailoring of patient-oriented cancer immunotherapy in concordance with the eventful and changeful dynamics of microenvironment, which will help determine the timing and dosing of the therapeutic schedule. Another phase III trial revealed that the concurrent chemoradiotherapt with liposomal tecemotide vaccine (NCT00409188) improved the survival to 9 months, which hints at the importance of the timing of combinatorial therapy (101).

## The modulation of gene therapy

The creation of novel cancer therapies continues to be hampered by innate and acquired drug resistance (99). Additionally, the short-term effectiveness of traditional therapies like chemotherapy may be compromised. Recent years have seen a rise in interest in gene therapy modification as a method of making tumor cells more drug-responsive. One of the most promising methods is to employ RNA to inhibit the production of certain proteins implicated in tumor resistance. On the basis of tumor abnormalities, silencing RNAs (siRNAs) or long non-coding RNAs (lncRNAs) have been transfected into tumor cells with encouraging results (102, 103). SiRNA administration has been notably investigated as a sensitizing treatment and a gene knockdown technique to reduce the expression of genes associated with cell growth and death. The signaling system, target of rapamycin (mTOR), which controls cell growth and metabolism by inhibiting apoptosis, has been extensively studied (104). The previous studies have revealed that due to their highly positive charge, serene encapsulation in polyplexes allows for high transfer rates. A current nanodrug delivery system exhibits exceptional endosomal escape ability, enabling siRNA delivery to the cytosol (105–107).

Furthermore, to enhance the effectiveness of treatments and prevent resistance mechanisms of tumors, it is imperative to restrict the development and metastatic capabilities of lung cancer while also sensitizing tumor cells. One of the most significant processes in NSCLC is the epithelial-mesenchymal transition (EMT). EMT is the process through which epithelial cells change into mesenchymal cells by losing their adherence and gaining the ability to differentiate and regenerate (108). This process raises the risk of tumor development and metastatic dissemination in cancer patients. Therefore, EMT must be regulated. By attaching to the 3’ untranslated region (UTR) of the target mRNA, microRNA (miRNA), a single-stranded short non-coding RNA, can be used to regulate gene expression (108). This stops the loss of epithelial cell-specific markers and proteins. Conversion can also be thwarted by other kinds of genetic material. In a prior work, siRNA was enclosed in an antibody-conjugated gelatin nanoparticle to block the expression of AXL. They were able to increase the tumor suppressor activity of the p53 pathway, decrease the expression of EMT proteins, and decrease the activity of mTOR.

In addition to inhibiting the expression of certain proteins, repair of the genome has become an increasingly popular treatment option for non-small cell lung cancer (NSCLC). By using CRISPR/Cas9 technology, it is possible to edit genes and open a wide range of possibilities (108). The method primarily involves the use of single-guide RNA-directed Cas9. As a result of the enzyme cleaving the DNA at the point of interest, a modification, deletion, or replacement of the DNA sequence can be accomplished. In light of this, the CRISPR/Cas9 system is widely applicable to the knockout of oncogenes and the study of genes associated with tumor suppression as well as genes associated with resistance.

## Conclusion

In summary, advances in nanotechnology have led to a plethora of treatment options for NSCLC. Combining nanomedicine with therapy has resulted in substantial improvements in both clinical benefit and toxicity. The objectives of nanodelivery systems include achieving optimal drug concentration in target cell populations, managing drug release, and improving long-term effects. There exist cutting-edge therapeutic methods such as siRNA, mRNA, and gene editing that have demonstrated effectiveness as anti-cancer techniques. These methods have been seamlessly integrated with nanotechnology and are expected to be made available to patients in the near future due to parallel advancements. Nevertheless, due to a lack of comprehensive understanding of the specific interactions between nanoparticles and biomolecules, designing effective trials is challenging. Moreover, further research is necessary to fully comprehend the issue of tumor nanoparticle permeability caused by insufficient EPR delivery efficiency.

## Author contributions

QL had full access to all of the data in the manuscript and take responsibility for the integrity of the data and the accuracy of the data analyses. All authors read and approved the final manuscript. Concept and design: All authors. Acquisition, analysis, or interpretation of the data: All authors. Drafting of the manuscript: LP, QX, SY, YZ, HW, YL, and QL. Critical revision of the manuscript for important intellectual content: LP, QX, LC, YH, JY, KP, and QL. Supervision: LP and QL. All authors contributed to the article.

## Glossary

**Table:**

ACS	American Cancer Society
AEs	adverse events
anti-PD-L1	anti-programmed cell death ligand 1
APC	antigen-presenting cells
ATP	adenosine-triphosphate
CSF1R	colony-stimulating factor 1 receptor
DCs	dendritic cells
DDS	drug delivery system
EGFR	epidermal growth factor receptor
EGFR-TKIs	EGFR-tyrosine kinase inhibitors
HDL	high-density lipoprotein
HMGB1	high mobility group protein B1
HSP90	heat shock protein 90
ICD	immunogenic cell death
ICIs	immune checkpoint inhibitors
IgG	immunoglobulin G
lncRNA	long-non-coding RNA
MNPs	magnetic nanoparticles
NK cell	natural killer cell
NIR	near-infrared
NSCLC	non-small cell lung cancer
MAGE-A3	melanoma-associated antigen A3
MDSCs	myeloid-derived suppressor cells
miRNA	microRNA
MUC1	mucinous glycoprotein-1 protein
mTOR	target of rapamycin
OS	overall survival
PAHs	polycyclic aromatic hydrocarbons
PTT	photothermal therapies
SCLC	small cell lung cancer
sHDL	high-density synthetic lipoprotein
siRNA	silencing RNA
SIRPα	signal-regulatory protein-α
TAA	tumor-associated antigens
TAMs	tumor-associated macrophages
TME	tumor microenvironment
Tregs	regulatory T cells
TLR	Toll-like receptor
TLR7	Toll-like receptor 7
TRAIL	tumor necrosis factor-related apoptosis inducing ligand
UTR	untranslated region
VEGF	vascular endothelial growth factor
WHO	World Health Organization
